# A prospective CSFV-PCV2 bivalent vaccine effectively protects against classical swine fever virus and porcine circovirus type 2 dual challenge and prevents horizontal transmission

**DOI:** 10.1186/s13567-023-01181-x

**Published:** 2023-07-11

**Authors:** Jing-Yuan Chen, Chi-Ming Wu, Min-Yuan Chia, Chienjin Huang, Maw-Sheng Chien

**Affiliations:** 1 Department of Veterinary Medicine, School of Veterinary Medicine, National Taiwan University, No. 1, Sec 4, Roosevelt Road, Taipei, 10617 Taiwan; 2 Graduate Institute of Veterinary Pathobiology, College of Veterinary Medicine, National Chung Hsing University, 145 Xingda Road, Taichung, 40227 Taiwan; 3 Department of Veterinary Medicine, College of Veterinary Medicine, National Chung Hsing University, 145 Xingda Road, Taichung, 40227 Taiwan; 4 Present Address: Graduate Institute of Microbiology and Public Health, College of Veterinary Medicine, National Chung Hsing University, 145 Xingda Road, Taichung, 40227 Taiwan

**Keywords:** classical swine fever, porcine circovirus-associated disease, bivalent vaccine, dual CSFV-PCV2 challenge trial, humoral and cellular immunity

## Abstract

Classical swine fever virus (CSFV) infection leading to CSF outbreaks is among the most devastating swine diseases in the pig industry. Porcine circovirus type 2 (PCV2) infection, resulting in porcine circovirus-associated disease (PCVAD), is also a highly contagious disease affecting pig health worldwide. To prevent and control disease occurrence, multiple-vaccine immunization is necessary in contaminated areas or countries. In this study, a novel CSFV-PCV2 bivalent vaccine was constructed and demonstrated to be capable of eliciting humoral and cellular immune responses against CSFV and PCV2, respectively. Moreover, a CSFV-PCV2 dual-challenge trial was conducted on specific-pathogen-free (SPF) pigs to evaluate vaccine efficacy. All of the vaccinated pigs survived and showed no clinical signs of infection throughout the experimental period. In contrast, placebo-vaccinated pigs exhibited severe clinical signs of infection and steeply increased viremia levels of CSFV and PCV2 after virus challenge. Additionally, neither clinical signs nor viral detections were noted in the sentinel pigs when cohabitated with vaccinated-challenged pigs at three days post-inoculation of CSFV, indicating that the CSFV-PCV2 bivalent vaccine completely prevents horizontal transmission of CSFV. Furthermore, conventional pigs were utilized to evaluate the application of the CSFV-PCV2 bivalent vaccine in field farms. An adequate CSFV antibody response and a significant decrease in PCV2 viral load in the peripheral lymph nodes were observed in immunized conventional pigs, suggesting its potential for clinical application. Overall, this study demonstrated that the CSFV-PCV2 bivalent vaccine effectively elicited protective immune responses and the ability to prevent horizontal transmission, which could be a prospective strategy for controlling both CSF and PCVAD in commercial herds.

**Supplementary Information:**

The online version contains supplementary material available at 10.1186/s13567-023-01181-x.

## Introduction

Porcine circovirus type 2 (PCV2) infection is the main aetiology of porcine circovirus-associated disease (PCVAD), which includes PCV2-systemic disease (PCV2-SD, substitution of post-weaning multisystemic wasting syndrome), PCV2-subclinical infection (PCV2-SI), PCV2-reproductive disease, and porcine dermatitis and nephropathy syndrome, and PCVAD has been one of the most prevalent swine viral diseases since it was first reported in the 1990s [1]. Vaccination is the primary strategy to minimize synergistic or sequential complications of concurrent infections and to reduce economic losses caused by PCVAD. However, current PCV2 vaccines are illustrated as “leaky vaccines”, meaning they can elicit protective efficacy against severe clinical signs and reduce viral replication and viremia levels but may not invoke sufficient immunity to eliminate the virus in infected pigs [2]. Consequently, PCV2-SI is a widespread global issue.

Classical swine fever virus (CSFV) infection leading to classical swine fever is one of the most contagious and devastating swine viral diseases affecting the pig industry in endemic countries. In contrast to the eradication policy adopted in most European Union countries, vaccination with live attenuated CSFV or a subunit vaccine is widely used in several Asian countries [3]. However, many unpredictable factors are believed to influence the efficacy of both live attenuated vaccines and subunit vaccines, such as the inability to maintain a cold temperature during transportation or storage and the fluctuation of batch manufacturing stability. In addition, varied vaccination programs and concurrent infection of other pathogens may substantially impact vaccine efficacy [4–7]. In vitro studies in porcine alveolar macrophages revealed that the infection and replication of live attenuated CSF virus (LPC strain) are compromised when concurrently infected with PCV2 [8]. In addition, in vivo studies have demonstrated that the interference of PCV2 infection on specific-pathogen-free (SPF) pigs or PCV2-SI in field pig farms may impact live attenuated CSF vaccine-induced immunity and vaccine efficacy [9, 10].

Many published studies report the concurrent infection of PCV2 with other viral pathogens, such as CSFV, porcine reproductive and respiratory virus, porcine parvovirus, swine influenza virus, pseudorabies virus, porcine epidemic diarrhoea virus, and torque teno sus virus in pigs [7, 11, 12]. Among these infectious pathogens coinfected with PCV2, the concurrent infection of PCV2 and CSFV may vary from 13.06% to 73.90% in commercial herds in different countries [11–13]. Although PCV2 vaccines have been utilized widely for decades, PCV2-SI is still predominant in endemic areas, suggesting that PCV2 infection could be a risk factor for the prevention and control of CSF [7, 12, 14]. Since CSF has a substantial economic impact on the pig industry, it is one of the notable swine viral diseases listed in the World Organization for Animal Health [3]. Prophylactic vaccination against CSFV is crucial for disease control or to even eliminate the pathogens. Accordingly, the development of novel bivalent vaccines may be one of the most effective approaches to minimizing the impact of PCV2-SI and preventing the outbreak of CSF. In this study, several animal trials were conducted to demonstrate that a CSFV-PCV2 bivalent vaccine could elicit antigen-specific antibody responses and interferon-γ (IFN-γ)-secreting cells in immunized animals. In addition, under the interference of PCV2 concurrent infection, the bivalent vaccine could provoke protective efficacy against a highly virulent CSFV challenge and completely restrict viral horizontal transmission among pigs, suggesting the potential of this CSFV-PCV2 bivalent vaccine in clinical application.

## Materials and methods

### Animals

The ICR mice were purchased from BioLasco Taiwan Co., Ltd. for evaluation of CSFV-PCV2 bivalent vaccine-induced humoral and cellular immune responses. SPF pigs (CSFV antigen/antibody-negative, PCV2 antigen/antibody-positive) were purchased from Animal Technology Laboratories, Agricultural Technology Research Institute, Miaoli, Taiwan, to evaluate CSFV-PCV2 bivalent vaccine-induced immunity. Conventional pigs were purchased from a continuous flow production pig farm located in Taichung, Taiwan. All animals in the study were fed ad libitum and raised in an isolated animal experimental facility. All animal trials and experimental procedures were reviewed and approved by the Institutional Animal Care and Use Committee (IACUC) of National Chung Hsing University under IACUC Approval number 103-45.

### Vaccines

The bivalent vaccine was composed of a baculovirus-expressed CSF-E2 subunit protein and a PCV2 capsid subunit protein (PCV2b Taiwan YL isolate, GenBank accession number: AY885225) that formed viral-like particles (Additional file 1) emulsified with a w/o/w adjuvant (Montanide ISA-201, SEPPIC, France) in an equal ratio. The baculovirus-expressed PCV2 capsid protein was purified by size exclusive chromatography and validated by transmission electron microscopy according to a previously published report [15]. Normal saline (0.9%) was utilized as a placebo to compare vaccine-induced immunity. The bivalent vaccine was formulated with the w/o/w adjuvant containing 45 µg of CSFV-E2 protein and 45 µg of PCV2/Cap protein per dose.

### Experimental design and sample collection

In the mouse trial, 10 6-week-old ICR mice were randomly assigned to two groups. The mice were intraperitoneally immunized (0.5 mL/dose) with the bivalent vaccine (*n* = 5) and placebo (*n* = 5) at 6 and 8 weeks of age, according to the prime-boost strategy (Table 1). Serum samples were collected at 12 weeks of age to evaluate the antigen-specific antibody level. All mice were euthanized at 14 weeks of age, and the splenocytes were isolated for an antigen-specific IFN-γ secreting cell enzyme-linked immunospot (ELISpot) assay.

**Table 1:** **Groups of ICR mice and vaccination program**

Groups and vaccines	Immunization
CSFV-PCV2 bivalent vaccine (*n* = 5)	Intraperitoneally immunized (0.5 mL/dose) two doses at 6, 8 weeks of age
Placebo (*n* = 5)	Intraperitoneally immunized (0.5 mL/dose) two doses at 6, 8 weeks of age

In the SPF pig trial, 12 6-week-old SPF pigs were randomly allocated to three groups and immunized with two doses (2 mL/dose) of CSFV-PCV2 bivalent vaccine (Group A, *n* = 4) and two doses (2 mL/dose) of placebo (Group B, *n* = 4) at 6 and 8 weeks of age. At 10 weeks of age, the peripheral blood mononuclear cells (PBMCs) of the pigs in Groups A and B were isolated to evaluate the vaccination-induced antigen-specific immune response using an ELISpot assay. The pigs in Groups A and B were challenged with 1.5 × 10^6^ TCID_50_ (50% tissue culture infectious dose) of CSFV (ALD strain) and 1 × 10^5^ TCID_50_ of PCV2 (PCV2a CYC08 strain) at 12 weeks of age. The pigs in Group C were nonvaccinated and nonchallenged as sentinel pigs and transferred to cohabitate with Group A at 3 days post-challenge (dpc) to detect whether there was virus shedding from the Group A pigs (Table 2). After challenge, clinical signs, including agility, appetite, excretion, respiratory rate, gaits, and body condition score (1–3 levels, 1: normal, 2: mild, 3: severe), and body temperature were recorded from 3 days pre- and post-challenge. The average daily weight gain (ADWG) was calculated pre-challenge (6–12 weeks of age) and post-challenge (12–15 weeks of age). Serum samples were collected at 0, 1, and 3 weeks post-challenge, and the antigen-specific antibody level and serum viral load were monitored. The Group B pigs were euthanized at 13 weeks of age (1 week post-challenge) due to severe clinical signs and weakness, whereas the pigs in Groups A and C were euthanized at 15 weeks of age.

**Table 2:** **Groups of SPF pigs and vaccination-challenge schedules**

Groups^a^	Vaccines^a^	Immunization schedules	Challenge^b^	Cohabitation^b^
A (*n* = 4)	CSFV-PCV2 bivalent vaccine	Two doses at 6, 8 weeks of age	CSFV and PCV2	Contact with Group C at 3 dpc
B (*n* = 4)	Placebo	Two doses at 6, 8 weeks of age	CSFV and PCV2	–
C (*n* = 4)	Sentinel pigs	Not vaccinated	Not challenged	Contact with Group A at 3 dpc

^a^Four-week-old SPF pigs (CSFV antigen and antibody-negative, PCV2 antigen negative/antibody-positive) were purchased from Animal Technology Laboratories, Agricultural Technology Research Institute. Pigs were randomly grouped and intramuscularly immunized with each vaccine (2 mL/dose) at the neck behind the ear twice at 6 and 8 weeks of age.

^b^Pigs in Groups A and B were challenged intramuscularly with 1.5 × 10^6^ TCID_50_ of CSFV (ALD strain) and 1 × 10^5^ TCID_50_ of PCV2 (CYC08 strain) at 11 weeks of age.

To evaluate the efficacy of the CSFV-PCV2 bivalent vaccine in clinical application, ten 3-week-old conventional piglets were randomly divided into two groups. Piglets in Group D were immunized with one dose (2 mL/dose) of CSFV-PCV2 bivalent vaccine at 4 weeks of age, and piglets in Group E were immunized with the placebo at the same age. Both Group D and Group E piglets were challenged with 1 × 10^5^ TCID_50_ PCV2 at 8 weeks of age and sacrificed at 12 weeks of age. Serum samples were collected at 4, 8, 9, 10, 11, and 12 weeks of age for antigen-specific antibody level analysis and screening of viral load. The submandibular, hilar, mesenteric and inguinal lymph nodes were collected for viral load screening and pathological analysis.

### Evaluation of humoral immunity

To detect the CSF-E2 and PCV2 bivalent subunit vaccine-induced antibody response, serum samples were collected and analysed by enzyme-linked immunosorbent assays (ELISA) to assess antibody levels. An SLK105 kit (BioChek BV, Reeuwijk, The Netherlands) was utilized to evaluate the PCV2-specific antibody level according to the manufacturer’s protocol, and the antibody level was expressed as the sample to positive (S/P) ratio. Serum samples with an S/P ratio greater than 0.50 were considered to be positive. An IDEXX CSFV Ab test kit (IDEXX Laboratories Inc., Liebefeld, Switzerland) was used to analyse CSFV-specific antibody titers in the serum, and the results were expressed as the blocking percentage. According to the manufacturer, serum samples with a blocking percentage greater than 40% are positive. In addition, the specific neutralizing antibody (NA) against CSFV (LPC strain) was conducted according to the diagnostic manual of the World Organization for Animal Health (WOAH) [16]. The NA level was subjected to log_2_ transformed analysis. According to Terpstra et al. and van Oirschot, an NA level greater than 1:32 in the tested pigs was considered adequate to protect individual pigs and prevent virus transmission in the population [17, 18].

### Detection of virus-specific IFN-γ secreting cells

To evaluate vaccine-induced cellular-mediated immunity, the ELISpot assay was performed according to the manufacturer’s instructions to measure the number of antigen-specific IFN-γ secreting cells in animals (SEL485 and SEL985, R&D systems, Minneapolis, MN, USA). Briefly, for a 96-well PVDF microplate, 100 µL of PBS-diluted IFN-γ capture antibody was loaded and incubated at 4 °C overnight. Before the onset of the ELISpot assay, wash the plate, block the membrane with 200 µL blocking buffer for 2 h and rinse with RPMI 1640 medium. In the mouse trial, 5 × 10^5^ mouse splenocytes were suspended in 100 µL of RPMI 1640 medium and treated with 1 multiplicity of infection (MOI) of CSFV (LPC strain) and 1 MOI of PCV2 (PCV2a CYC08 strain). In the SPF pig trial, 5 × 10^5^ pig PBMCs were isolated and treated with 1 MOI of CSFV (LPC strain), 1 MOI of PCV2 (PCV2a CYC08 strain), 10 µg of CSF-E2 subunit protein, and 10 µg of PCV2 capsid subunit protein. After 24 h of incubation at 37 °C in a 5% CO_2_ incubator, the cells and culture medium were removed from the 96-well plate and washed with 0.05% Tween 20 in PBS. Add 100 µL of detection antibody to each well and incubate overnight at 4 °C. The ELISpot blue colour module (SEL002, R&D systems) was used for spot colour development. A positive reaction is indicated by the blue spots, and each spot represents an antigen-specific IFN-γ-secreting cell.

### Nucleotide extraction and virus detection

Serum viral DNA was extracted using a DNeasy Blood and Tissue kit (69509, Qiagen GmbH, Hilden, Germany), and serum viral RNA was extracted using a NucleoSpin^®^ RNA kit (740955.50, Macherey-Nagel GmBH & Co. KG, Duren, Germany) according to the manufacturer’s instructions. The total nucleotides in tissue samples from pigs were extracted using a taco™ Nucleic Acid Automatic Extraction System (GeneReach Biotechnology Corp., Taichung, Taiwan) and a taco™ DNA/RNA Extraction kit (atc-d/rna, GeneReach Biotechnology Corp.) according to the manufacturer’s instructions. The RNA was reverse transcribed using an iScript cDNA synthesis kit (1708891, Bio-Rad, Hercules, CA, USA) according to the manufacturer’s procedures. The real-time PCR method using SYBR Green I was used to detect the CSFV 5′ UTR gene (modified from Hoffmann et al.) and PCV2 ORF1 gene with specific primer sets (CSFV-forwards: 5′-CACACCACGTGATGGGAGTA-3′, CSFV-reverse: 5′-CTCCATGTGCCATGTACAGC-3′; PCV2-forwards: 5′-AAAAGCAAATGGGCTGCTAA-3′, PCV2-reverse: 5′-TGGTAACCATCCCACCACTT-3′) [19, 20]. For each 20 µL reaction, primers and cDNA/DNA samples were added to 2 × iTaq universal SYBR green supermix (1,725,121, Bio-Rad) to a final concentration of 0.2 µM along with 5 µL of cDNA/DNA template. The amplification reaction was carried out as follows: incubation at 95 °C for 5 min and 50 cycles of 95 °C for 10 s, 60 °C for 10 s, and then 72 °C for 10 s. After amplification, a melting curve analysis was performed to verify the specificity. Real-time PCR was performed using the CFX connect™ real-time PCR detection system (Bio-Rad), and the threshold cycle values (Ct) of each reaction were calculated using Bio-Rad CFX manager version 3.1 (Bio-Rad). The quantification data were log_10_ transformed for analysis. A threshold of serum PCV2 viral load at 10^2^ copies/µL was set according to a previous study [9].

### Pathological and immunohistochemical (IHC) analysis

The lymph nodes were harvested and fixed with 10% neutral formalin. Paraffin-embedded tissue sections were subjected to haematoxylin and eosin (H&E) staining and IHC staining for the PCV2 antigen. A PCV2-specific monoclonal antibody (M.11.PCV. I36A9, Ingenasa, Madrid, Spain) was used for IHC staining according to previously reported procedures [9]. To evaluate the PCV2-specific antigen density score, 10 randomly selected intact germinal centre images were captured under a 200× vision field for each lymph node (40 images per pig). The pixels of DAB staining in each image were quantitated with ImageJ software version 1.53e with an IHC toolbox and colour counter plugin according to a previous study and the data were recorded as the IHC density score [21].

### Statistical analysis

Data analysis was performed using R software version 4.2.2 (The R Foundation, Vienna, Austria). The Kruskal–Wallis test and pairwise Wilcoxon rank-sum test were used for statistical analysis, and differences with a *p* value less than 0.05 were considered statistically significant.

## Results

### Evaluation of the CSFV-PCV2 bivalent vaccine-induced immune response in mice

**Figure 1:**
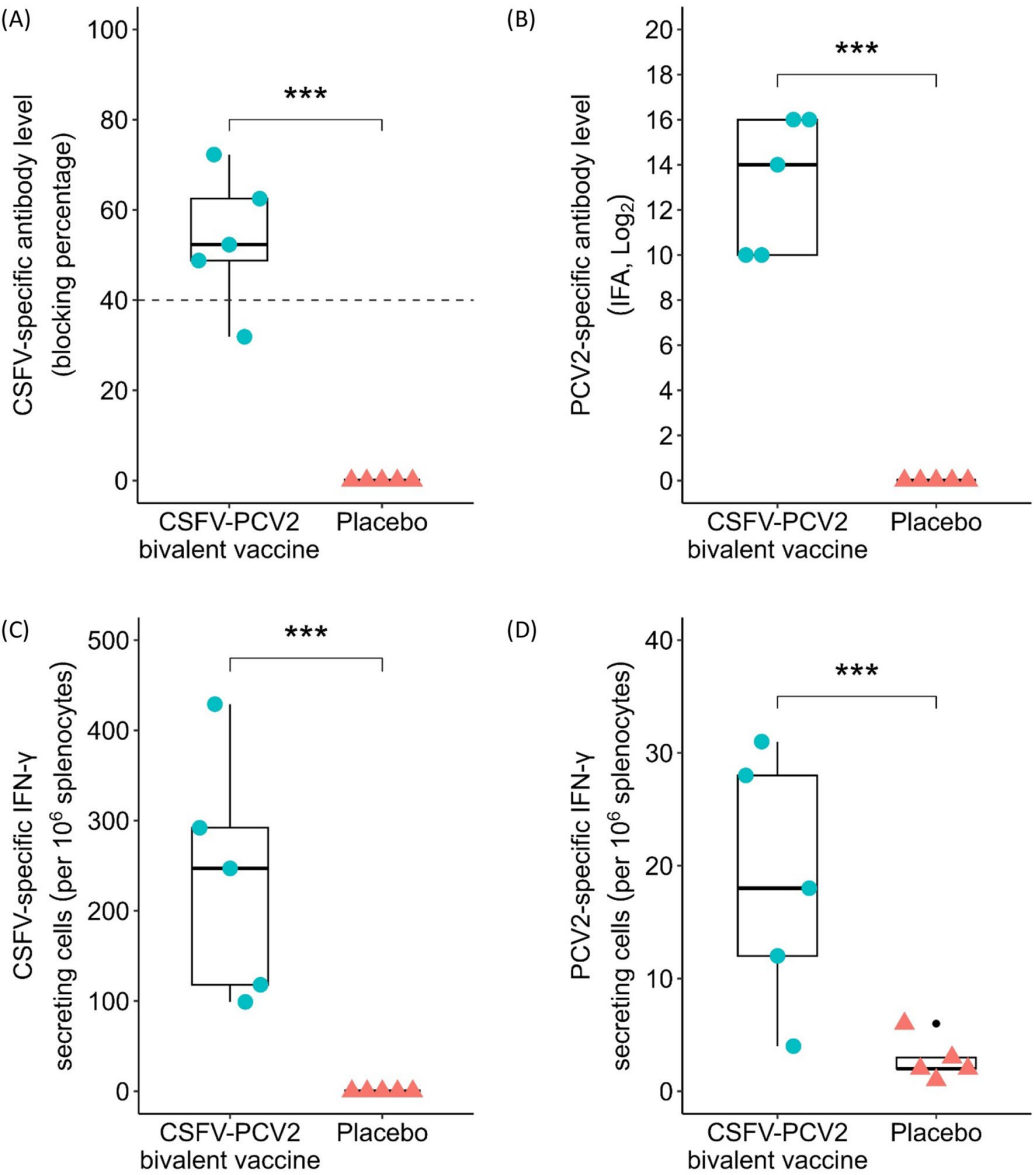
**CSFV-PCV2 bivalent vaccine provokes immune responses in a mice model.** ICR mice were intraperitoneally immunized (0.5 mL/dose) with CSFV-PCV2 bivalent vaccine (*n* = 5) or placebo (*n* = 5) at 6 and 8 weeks of age. Serum samples were collected at 12 weeks of age to evaluate CSFV-specific (**A**) or PCV2-specific (**B**) antibody levels. Splenocytes were isolated at 14 weeks of age to evaluate the number of CSFV-specific (**C**) and PCV2-specific (**D**) IFN-γ-secreting cells by ELISpot assay. Data are presented in a box plot, and each spot indicates an individual mouse. The Kruskal–Wallis test was used for statistical analysis, and differences were considered statistically significant at a *p* value < 0.05.

In the mouse trial, ICR mice were immunized with the CSFV-PCV2 bivalent vaccine or placebo at 6 and 8 weeks of age. The CSFV-PCV2 bivalent vaccine elicited both CSF-specific (53.54 ± 6.80%) (Figure 1A) and PCV2-specific (13.20 ± 1.36 log_2_) (Figure 1B) antibody responses in immunized mice at 12 weeks of age. To evaluate the bivalent vaccine-induced cellular immunity, mouse spleen cells were isolated at 14 weeks of age and subjected to an ELISpot assay with stimulation of 1 MOI of CSFV or 1 MOI of PCV2. The results indicated that the CSFV-PCV2 bivalent vaccine induced both CSFV-specific IFN-γ-secreting cells (237.00 ± 60.50 cells/10^6^ splenocytes) (Figure 1C) and PCV2-specific IFN-γ-secreting cells (18.60 ± 5.00 cells/10^6^ splenocytes) (Figure  1D). These results showed that the CSFV-PCV2 bivalent vaccine may induce humoral and cellular immune responses in a laboratory animal model.

### The CSFV-PCV2 bivalent vaccine provokes humoral and cellular immune responses in pigs

**Figure 2:**
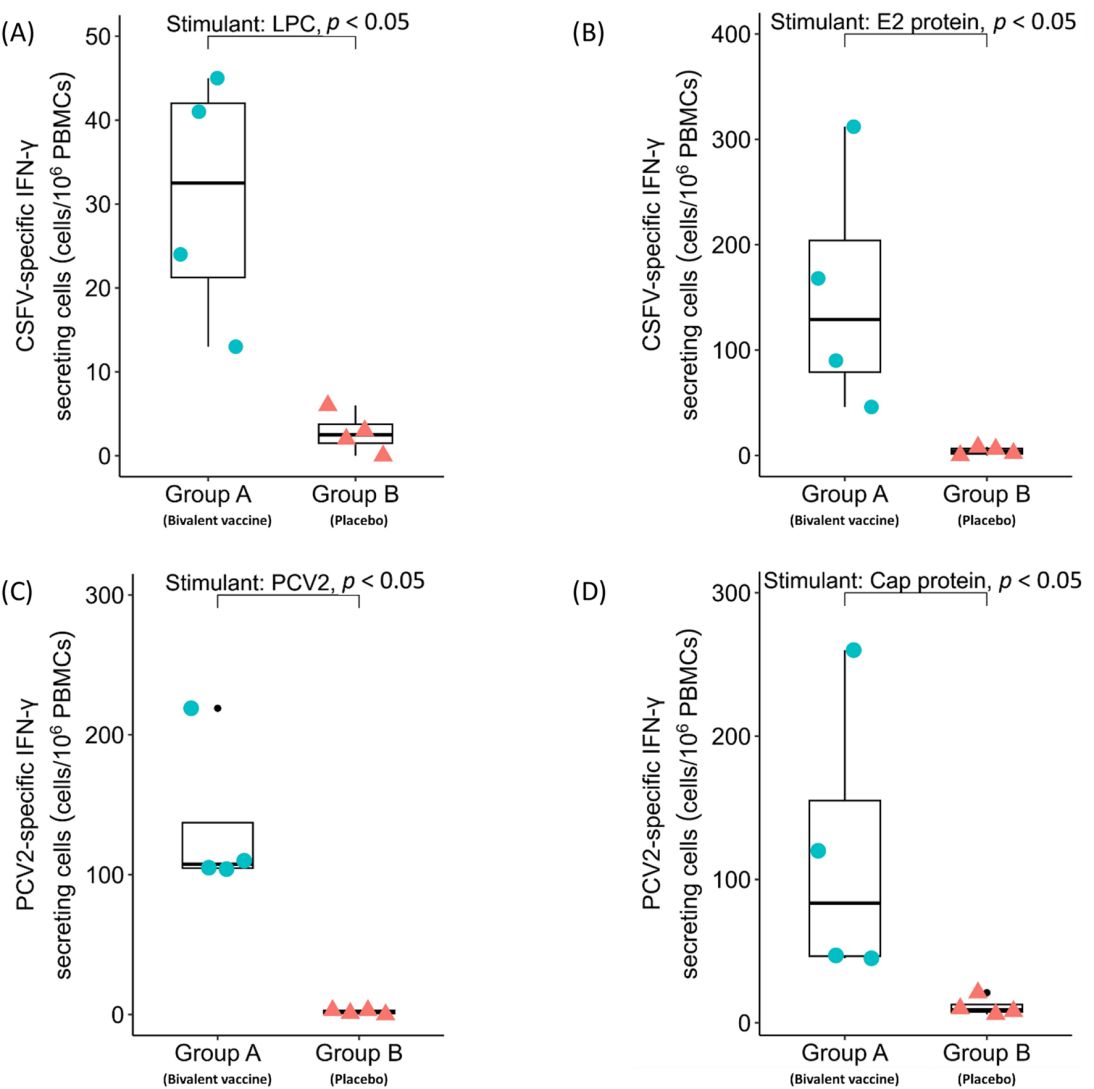
**CSFV-PCV2 bivalent vaccine provokes antigen-specific cellular immune response in SPF pigs.** SPF pigs were immunized with two doses of CSFV-PCV2 bivalent vaccine (Group A, *n* = 4) or placebo (Group C, *n* = 4) at 6 and 8 weeks of age. PBMCs were isolated and stimulated with the viruses (CSFV LPC strain and PCV2) and subunit proteins (CSF-E2 and PCV2 capsid protein). The antigen-specific IFN-γ secreting cells for 1 MOI LPC virus (**A**), 10 µg of CSF-E2 subunit protein (**B**), 1 MOI PCV2 virus (**C**), and 10 µg of PCV2 capsid protein (**D**) were calculated. Data are presented in a box plot, and each spot indicates an individual mouse. The Kruskal–Wallis test was used for statistical analysis, and differences were considered statistically significant at a *p* value < 0.05.

To evaluate CSFV-PCV2 bivalent vaccine-induced immunity, SPF pigs (CSFV antigen/antibody-negative, PCV2 antigen/antibody-positive) were immunized with CSFV-PCV2 bivalent vaccine (Group A) or placebo (Group B) at 6 and 8 weeks of age. Serum samples were collected, and the analysed vaccine elicited an antigen-specific immune response at 9 weeks of age. The results revealed that Group A pigs (58.79 ± 5.84%) showed significantly higher CSFV-specific antibody levels than Group B pigs (Additional file 2). In addition, at 10 weeks of age, PBMCs were isolated to evaluate the number of vaccine-induced antigen-specific IFN-γ-secreting cells. With the stimulation of CSFV (LPC strain) (Figure 2A) or CSF-E2 subunit protein (Figure 2B), pigs in Group A (LPC: 30.75 ± 7.47 cells/10^6^ PBMCs; CSF-E2: 154.00 ± 58.40 cells/10^6^ PBMCs) showed a significantly higher number of CSFV-specific IFN-γ secreting cells than Group B (LPC: 2.75 ± 1.25 cells/10^6^ PBMCs; CSF-E2: 3.65 ± 1.83 cells/10^6^ PBMCs).

Since all pigs were PCV2 antigen and antibody-positive before the beginning of the trial, there were no obvious dynamics of PCV2-specific antibody levels noted during the experimental period. However, the S/P ratio of PCV2-specific antibody levels was 1.90 ± 0.60 in bivalent vaccine-immunized Group A and 0.73 ± 0.56 in placebo-immunized Group B at 12 weeks of age. Since all of SPF pigs were kept in a high-containment animal biosecurity level II unit, the PCV2-specific antibody level was less likely to be induced by PCV2 contamination. The results suggested that the higher antibody level in Group A at 12 weeks of age may be provoked by CSFV-PCV2 bivalent vaccine immunization (Additional file 2). Moreover, with the stimulation of PCV2 (Figure 2C) or PCV2 capsid subunit protein (Figure 2D), the Group A pigs (PCV2: 134.50 ± 28.20 cells/10^6^ PBMCs; capsid protein: 118.00 ± 50.45 cells/10^6^ PBMCs) showed a significantly higher number of PCV2-specific IFN-γ secreting cells than the Group B pigs (PCV2: 1.50 ± 0.75 cells/10^6^ PBMCs; capsid protein: 11.25 ± 3.35 cells/10^6^ PBMCs). These results demonstrated that the CSFV-PCV2 bivalent vaccine induced an antigen-specific antibody response against CSFV. In addition, cellular immunity against both CSFV and PCV2 was detected in vaccine-immunized pigs.

### Complete protective efficacy was provoked against dual viral challenge and prevented virus transmission to sentinel pigs

**Figure 3:**
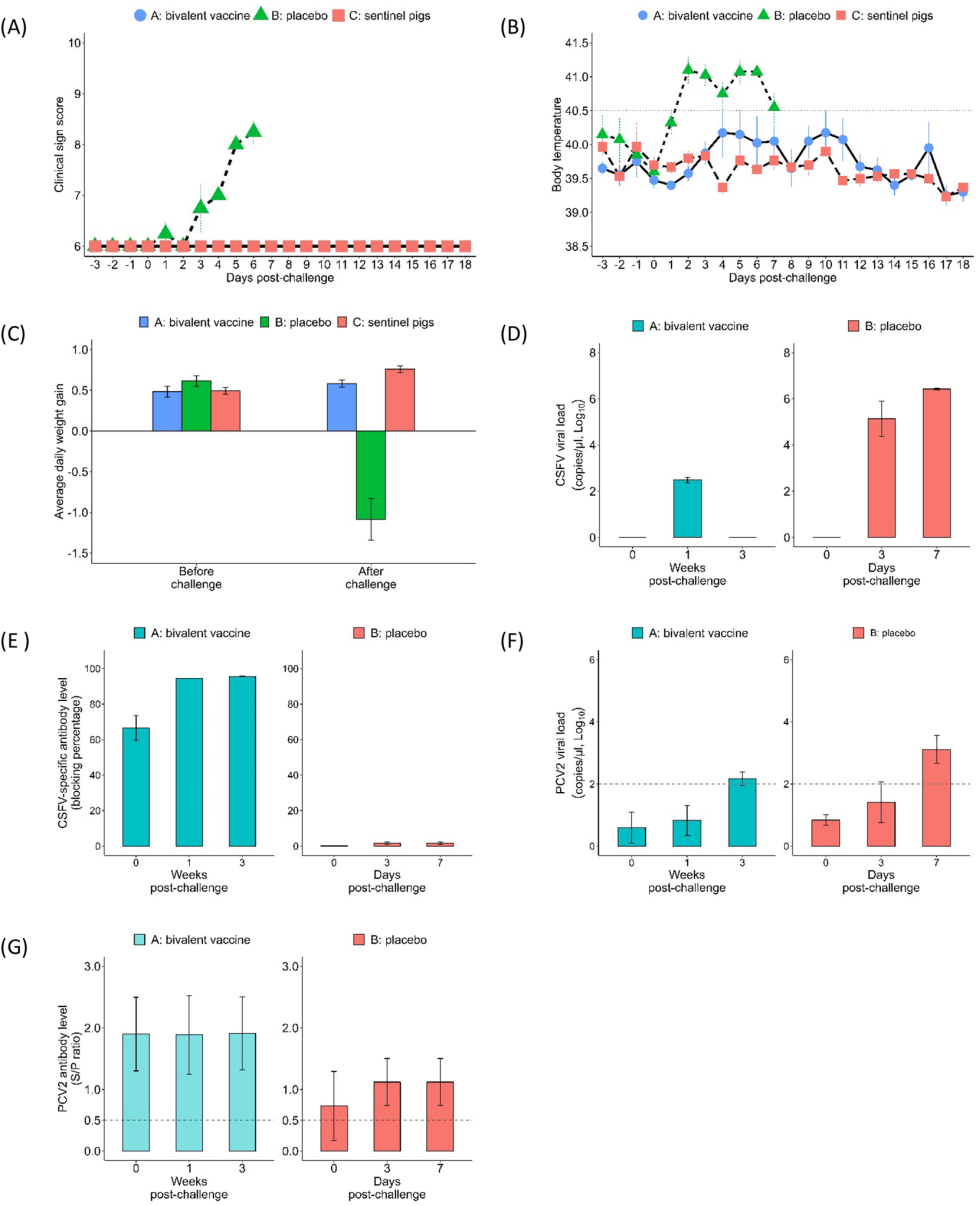
**Bivalent vaccine protected immunized pigs against CSFV and PCV2 virus challenge and prevented horizontal transmission.** SPF pigs immunized with CSFV-PCV2 bivalent vaccine immunization (Group A, *n* = 4) or placebo (Group B, *n* = 4) were challenged with 1.5 × 10^6^ TCID_50_ of CSFV (ALD strain) and 1 × 10^5^ TCID_50_ of PCV2 (CYC08 strain) at 12 weeks of age. Three days after challenge, the sentinel pigs (Group C, *n* = 4) were cohabitated with Group A to monitor virus horizontal transmission. Pigs in Group B were euthanized at 13 weeks of age (1 week post-challenge) due to severe clinical signs of infection and weakness, whereas pigs in Groups A and C were euthanized at 15 weeks of age. The clinical signs (**A**), body temperature (**B**), and average daily weight gain (**C**) were monitored and recorded as indicators for virus-induced disease. Serum samples were utilized for virus screening (**D**, **F**) and dynamic antibody levels (**E**, **G**). Data are presented as the mean ± standard error of the mean.

Four weeks after the immunization boost, the pigs in Groups A and B were challenged with 1.5 × 10^6^ TCID_50_ of CSFV (ALD strain) and 1 × 10^5^ TCID_50_ of PCV2 (PCV2a CYC08 strain) at 11 weeks of age. The sentinel pigs (Group C) were transferred to be in contact with Group A at 3 days post-challenge (Table 2). After the challenge, the clinical sign score of the Group B pigs steeply increased, and hyperpyrexia was noted at 2 dpc (Figures 3A and B). In addition, the ADWG of the Group B pigs significantly decreased after challenge (Figure 3C). All pigs in Group B were euthanized at 7 dpc due to severe clinical signs of infection and weakness, whereas all pigs in Group A survived the challenge and were euthanized at 14 weeks of age. In addition, a significantly high CSFV viral load was detected in Group B at 3 dpc (5.14 ± 0.76 copies/µL) and 7 dpc (6.42 ± 0.04 copies/µL) (Figure 3D). The increased CSFV viral load corresponded to the severe clinical sign score and hyperpyrexia, suggesting an acute CSFV infection. The sentinel pigs, Group C, showed unremarkable clinical signs and no detectable CSFV viral load during the experiment, indicating no horizontal transmission during cohabitation with Group A. Group A showed significantly high CSFV-specific antibody levels before (66.54 ± 6.98%) and after (95.65 ± 0.25% at 3 weeks post-challenge) the challenge, indicating that the bivalent vaccine provoked an immune response (Figure 3E). The Group B pigs showed a steeply increased PCV2 viral load at 7 dpc (3.11 ± 0.44 copies/µL) (Figure 3F) but lower PCV2-specific antibody levels (Figure 3G).

### Application of CSFV-PCV2 bivalent vaccine in conventional pigs

**Figure 4:**
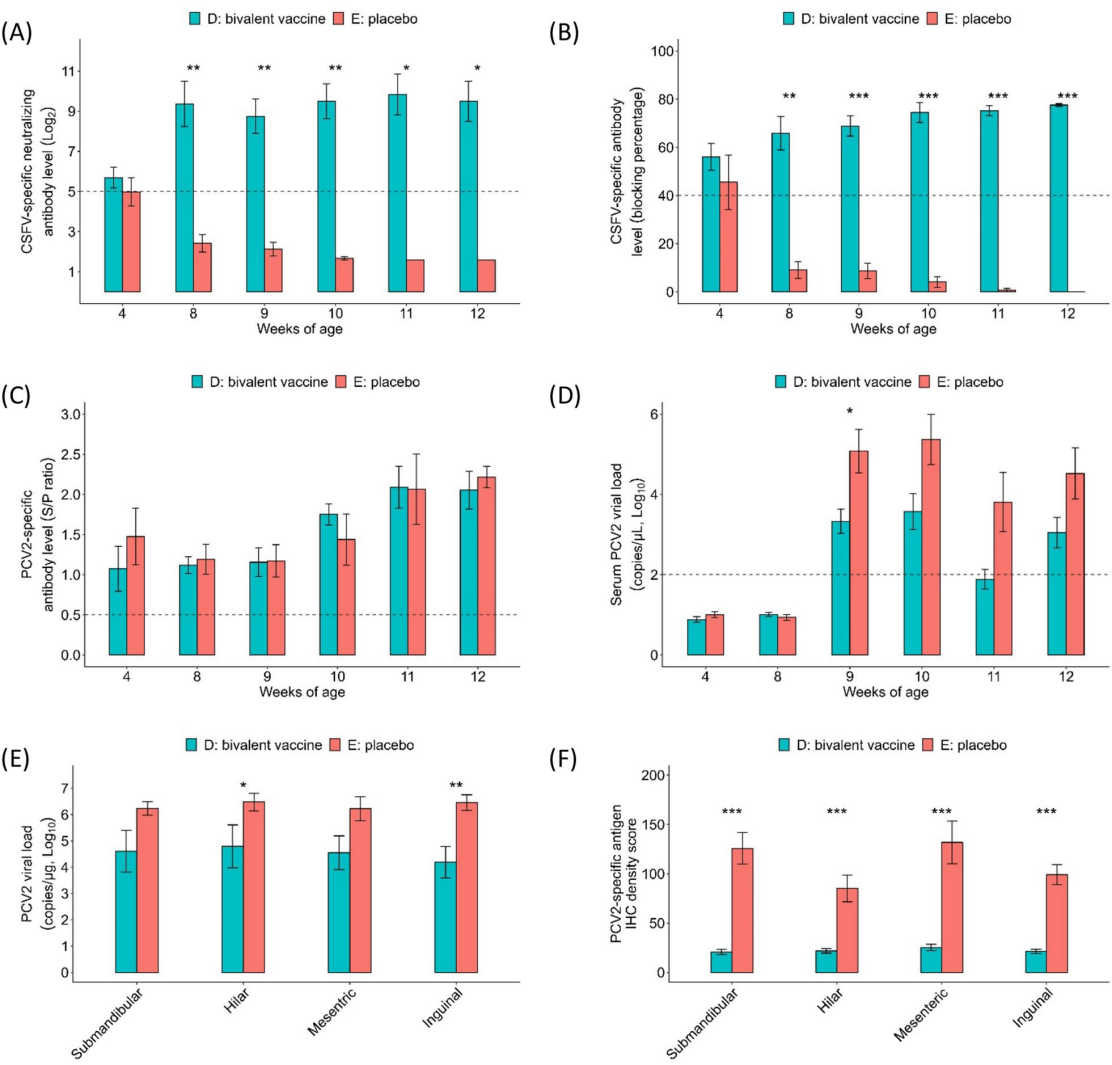
**Application of the CSFV-PCV2 bivalent vaccine to conventional pigs.** Conventional pigs were divided into two groups and immunized with one dose of CSFV-PCV2 bivalent vaccine (Group D, *n* = 5) and placebo (Group E, *n* = 5) at 4 weeks of age. All pigs were challenged with 1 × 10^5^ TCID_50_ of PCV2 at 8 weeks of age. The CSFV-specific antibody response was evaluated by neutralization assay (**A**) and ELISA (**B**). The serum PCV2-specific antibody level and PCV2 viral load were monitored by ELISA (**C**) and real-time PCR (**D**). All pigs were sacrificed at 12 weeks of age. The peripheral lymph nodes were subjected to viral load detection by real-time PCR (**E**) and IHC analysis (**F**). Data are presented as the mean ± standard error of the mean, and the Kruskal–Wallis test was used for statistical analysis. Differences were considered statistically significant at a *p* value < 0.05.

**Figure 5:**
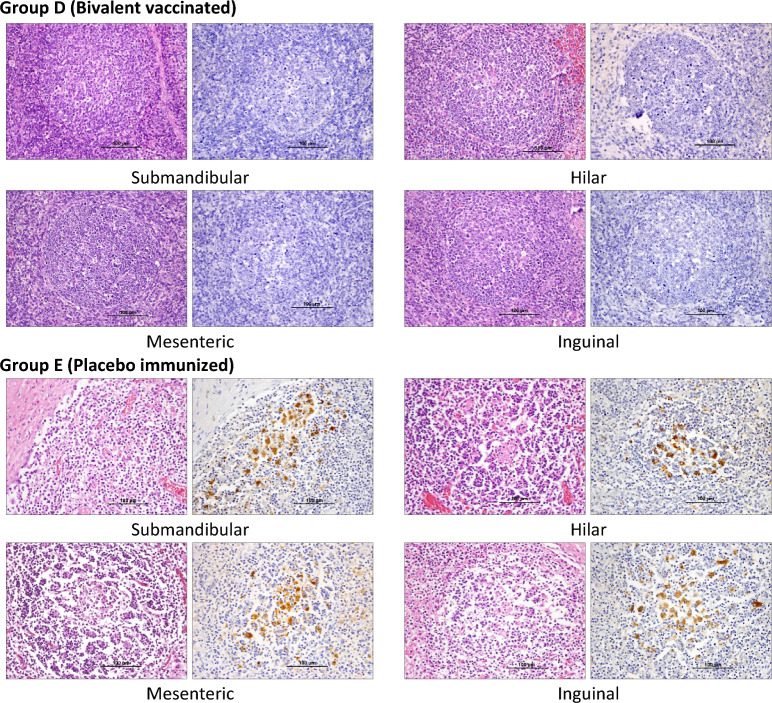
**Analysis of the histopathological changes in the peripheral lymph nodes after PCV2 challenge.** The lymph nodes from vaccinated (Group D) and placebo-immunized (Group E) pigs, including the submandibular, hilar, mesenteric, and inguinal lymph nodes were collected at 4 weeks after PCV2 challenge and were processed for H&E and IHC staining for the PCV2 antigen. (left: H&E staining, right: IHC staining specific for PCV2 capsid protein).

After confirming the protective efficacy of the CSFV-PCV2 bivalent vaccine in SPF pigs, the CSFV-PCV2 bivalent vaccine was further applied in conventional pigs. Conventional pigs were immunized with one dose of CSFV-PCV2 bivalent vaccine (D) or placebo (E) at 4 weeks of age and challenged with 1 × 10^5^ TCID_50_ of PCV2 at 8 weeks of age. Pigs in Group D showed a significantly higher CSFV-specific antibody response in the NA level (9.37 ± 1.13 log_2_ versus 2.42 ± 0.43 log_2_, Figure 4A) and ELISA (65.96 ± 6.98% versus 9.10 ± 3.52%, Figure 4B) results than pigs in Group E after immunization at 8 weeks of age and throughout the experiment. As a previous study reported [22], there was no significant difference in the dynamics of PCV2-specific antibody levels between Groups D and E (Figure 4C). However, the serum PCV2 viral load in Group E (5.08 ± 0.54 log_10_ copies/µL) steeply increased and was significantly higher than that in Group D (3.33 ± 0.30 log_10_ copies/µL) after PCV2 challenge at 9 weeks of age, suggesting artificial PCV2 infection of placebo-vaccinated pigs (Figure 4D). Moreover, Group E also showed significantly higher PCV2 viral load (Figure 4E) and PCV2-specific antigen density (Figure 4F) in all peripheral lymph nodes according to the IHC examination (Figure 5).

## Discussion

The use of vaccines to control PCVAD is one of the most effective and commonly accepted measures by swine breeders and veterinarians, and PCV2 vaccines are the largest selling and applied prophylactic vaccines in porcine husbandry. In addition to PCV2 vaccines, several other vaccines are required for conventional pigs during the entire production period. The live attenuated CSF vaccine has been widely utilized or made mandatory to control CSFV epidemics [3]. Despite this increased usage, the emergence of epidemic diseases such as African swine fever virus (ASFV) may lead to ineffective CSF vaccine application in ASFV-affected areas [23]. Therefore, the development of bivalent or multivalent vaccines combined with PCV2 vaccines may offer an alternative solution to avoid elevated stress from multi-immunization processes and the interference of concurrent infections via PCV2-SI in field farm applications. In this study, several animal models, including a mouse model and SPF and conventional pig models were used to conduct vaccination or vaccination-challenge trials to evaluate the safety and efficacy of a prospective CSFV-PCV2 bivalent vaccine (Table 3).

**Table 3:** **Groups of conventional pigs and vaccination-challenge schedules**

Groups^a^	Vaccines^a^	Immunization schedules	Challenge^b^
D (*n* = 5)	CSFV-PCV2 bivalent vaccine	One dose at 4 weeks of age	PCV2
E (*n* = 5)	Placebo	One dose at 4 weeks of age	PCV2

^a^Four-week-old conventional pigs were randomly divided into two groups and intramuscularly immunized with one dose (2 mL/dose) of CSFV-PC2 bivalent vaccine or placebo.

^b^Pigs in Groups D and E were challenged intramuscularly with 1 × 10^5^ TCID_50_ of PCV2 (CYC08 strain) at 8 weeks of age.

Before the pig immunization trials, a mouse model was used to evaluate the safety and induced immunity of the CSFV-PCV2 bivalent vaccine. There was no adverse effect noted in vaccinated animals, and no pathological change was found around the injected sites, demonstrating the safety of the CSFV-PCV2 bivalent vaccine. The results from the ELISpot assay showed that the mouse splenocytes exhibited an antigen-specific IFN-γ response after stimulation with 1 MOI of CSFV (LPC strain) and 1 MOI of PCV2 (PCV2a CYC08 strain). The CSFV glycoprotein E2, presenting as a homodimer on the viral surface, is crucial for virus entry into target cells and triggers the host immune response. Moreover, several epitopes on the CSF-E2 protein have been demonstrated to provoke neutralizing antibodies and trigger cellular immunity in several previous studies [24–26]. In addition, it has been suggested that provoked antigen-specific IFN-γ responses against PCV2 are crucial for pigs to prevent infection [27]. To gain better insight into the CSFV-PCV2 bivalent vaccine-induced IFN-γ response, recombinant proteins (10 µg of CSF-E2 subunit protein and 10 µg of PCV2 capsid protein) and viruses (1 MOI of CSFV and 1 MOI of PCV2) were utilized to determine the IFN-γ responses of PBMCs in the SPF pig model. The results showed that there was a comparable level of ELISpot response between the two recombinant proteins (CSF-E2: 154.00 ± 58.40; PCV2 capsid protein: 118.00 ± 50.45) and both viruses (CSFV: 30.75 ± 7.47, PCV2: 134.50 ± 28.20) in PBMCs from immunized pigs (Group A). In fact, regardless of whether the expressed antigen or virus was used for analysis, the results demonstrated that the bivalent vaccine-immunized group had significantly higher antigen-specific IFN-γ responses than the placebo-immunized group in the SPF pig experiment (Figure 2).

The efficacy of the CSFV-PCV2 bivalent vaccine was further evaluated in SPF and conventional pigs. Analysis of serum CSFV-specific antibody levels in Group A pigs showed significantly higher and more concentrated distribution than those of Group B pigs following immunization and exhibited a rapid increase at 1 week post-viral challenge. Screening of the serum CSFV viral load showed only a mild increase in CSFV levels in Group A pigs at 1 week post-challenge. However, no viral load was detected at 3 weeks post-challenge, suggesting the elimination of CSFV in bivalent vaccine-immunized pigs.

In contrast to the strong neutralizing antibody response against CSFV, there were limited PCV2-specific antibody levels in SPF pigs. In fact, serological investigations of commercial PCV2 vaccine-immunized pigs revealed varied antibody levels and viral loads in vaccinated pigs, suggesting that PCV2-SI possibly exists in herds. In this study, despite the limited induced antibody level, there was a distinct difference in the PCV2 viremia level between vaccine-immunized Group A and placebo-immunized Group B after PCV2 challenge. The serum PCV2 viral load steeply increased at 7 days post-challenge in Group B pigs (3.11 ± 0.44 copies/µL); however, the viral load was limited in vaccine-immunized Group A pigs (0.83 ± 0.49 copies/µL). This result suggested that the CSFV-PCV2 bivalent vaccine could elicit a protective immune response against PCV2 infection and viral replication.

Several published studies have demonstrated that the production of antigen-specific IFN-γ is crucial to prevent PCV2 and CSFV infection [28–30]. However, most previously developed CSFV subunit vaccines may not be able to induce adequate cellular immunity [5]. Since the E2 subunit protein is one of the main antigens that induces the cellular immune response in CSFV infection, studies have reported the combined expression of the E2 subunit and swine IFN-γ as an immunoadjuvant [3, 31]. Although the expression of the CSFV-E2 subunit protein along with recombinant swine IFN-γ may possibly enhance the cellular immune response, it may alter the immune homeostasis in immunized PCV-SI pigs. Several studies have demonstrated that the administration of IFN-γ to PCV2-infected cells may compromise immunity, enhance virus replication and lead to cell death [32–34]. In this study, the CSFV-PCV2 bivalent vaccine emulsified with Montanide ISA-201 adjuvant was confirmed to enhance the antigen-specific cellular immune response in animals [35]. Recently, a study demonstrated that an influenza virus haemagglutinin-based subunit protein vaccine may increase the frequency of viral-specific CD4^+^ T cells, which was correlated with higher humoral and cellular immunity [36]. The results of the ELISpot assay on mouse splenocytes and pig PBMCs (Figure 1 and Figure 2) showed significantly higher numbers of CSFV-specific and PCV2-specific IFN-γ-secreting cells, suggesting that the CSFV-PCV2 bivalent vaccine elicited antigen-specific IFN-γ secretion in vaccinated animals.

In addition to the SPF pig model, a PCV2 challenge trial was conducted to evaluate the efficacy of the CSFV-PCV2 bivalent vaccine on conventional pigs. The CSFV-PCV2 bivalent vaccine-immunized pigs (Group D) showed significantly higher CSFV-specific neutralizing antibody levels than placebo-vaccinated pigs in Group E at 4 weeks post-vaccination (8 weeks of age) and throughout the experimental period (Figure 4A and B). Following PCV2 challenge, the PCV2 viral load steeply increased in Group E and was significantly higher than that in Group D, suggesting that the status of PCV2-SD was identified in placebo-vaccinated and challenged pigs (Figure 4D). Furthermore, the analysis of the peripheral lymph nodes of pigs in Group E showed significantly higher PCV2 viral load and IHC staining scores, coordinating with the severe lymphoid depletion and degeneration under the microscopic examination in Group E (Figure 5). The challenge trial in conventional pigs demonstrated the potential application of the CSFV-PCV2 bivalent vaccine in field farms.

Concurrent infection is a more frequent occurrence than a single pathogen-induced disease in present-day field farms [37]. In most Asian countries, preventive vaccination is the primary strategy for CSF and PCV2 control. Herein, we evaluated the safety and efficacy of a novel CSFV-PCV2 bivalent vaccine using mouse and pig animal trials. The results demonstrated that the CSFV-PCV2 bivalent vaccine could provoke adequate protective efficacy against dual viral challenge and prevent virus horizontal transmission. Furthermore, the CSFV-PCV2 bivalent vaccine also elicited antigen-specific IFN-γ secreting cells in vaccinated animals that effectively improved the vaccine efficacy and circumvented the disadvantage of subunit vaccines. In summary, this study presents a prospective CSFV-PCV2 bivalent vaccine that can induce both humoral and cellular immunity against CSFV and PCV2 individual and dual challenge. Moreover, the efficacy of the CSFV-PCV2 bivalent vaccine was confirmed via a challenge trial in immunized conventional pigs, suggesting that the CSFV-PCV2 bivalent vaccine may serve as a prospective strategy for controlling CSF and PCVAD in commercial herds.

## Supplementary Information

**Additional file 1. Analysis of PCV2 capsid subunit protein-formed viral-like particles**. The viral-like particles of purified PCV2 capsid protein were negatively stained and analysed by transmission electron microscopy at 120 kV and 400 000 × . Scale bar is 20 nm.

**Additional file 2. Profile of PCV2 antigen and antibody levels as well as CSFV antigen and the dynamics of CSFV-specific antibodies in SPF pigs (Groups A, B, and C)**. The PCV2 and CSFV viral loads were detected by real-time PCR and expressed as copies/µL. The PCV2-specific antibody level was detected by an SLK105 kit (BioChek BV, Reeuwijk, The Netherlands), and the data were expressed as the S/P ratio. According to the kit’s protocol, serum samples with an S/P ratio greater than 0.50 were considered positive. In addition, the CSFV-specific antibody level was evaluated using the IDEXX CSFV Ab test kit (IDEXX Laboratories Inc., Liebefeld, Switzerland). The results were expressed as the blocking percentage, and according to the manufacturer, serum samples with a blocking percentage greater than 40% were considered positive.

## Data Availability

The datasets used and/or analysed during the current study are available from the corresponding author on reasonable request.

## Abbreviations

CSFV: classical swine fever virus
CSF: classical swine fever
PCV2: porcine circovirus type 2
PCVAD: porcine circovirus-associated disease
IFN-γ: interferon-γ
SPF: specific-pathogen-free
PCV2-SD: PCV2-systemic disease
PCV2-SI: PCV2-subclinical infection
IACUC: institutional animal care and use committee
ELISpot: enzyme-linked immunospot
PBMCs: peripheral blood mononuclear cells
TCID_50_: 50% tissue culture infectious dose
dpc: days post-challenge
ADWG: average daily weight gain
S/P ratio: sample to positive ratio
NA: neutralizing antibody
WOAH: World Organization for Animal Health
MOI: multiplicity of infection
Ct: threshold cycle value
IHC analysis: immunohistochemical analysis
H&E staining: haematoxylin and eosin staining
ASFV: African swine fever virus
