# Supplementary material for: A prospective CSFV-PCV2 bivalent vaccine effectively protects against classical swine fever virus and porcine circovirus type 2 dual challenge and prevents horizontal transmission
Source: Vet Res. 2023 Jul 11;54:57. doi: 10.1186/s13567-023-01181-x (PMC10337183; doi:10.1186/s13567-023-01181-x)
Supplement: Supplementary file 1 — Additional file 1. Analysis of PCV2 capsid subunit protein-formed viral-like particles. The viral-like particles of purified PCV2 capsid protein were negatively stained and analysed by transmission electron microscopy at 120 kV and 400 000 × . Scale bar is 20 nm. [file 13567_2023_1181_MOESM1_ESM.docx]

**Additional file 1 Analysis of PCV2 capsid subunit protein formed viral-like particle.** The viral-like particles of purified PCV2 capsid protein was negative stained and was analyzed by transmission electron microscope at 120 kV and 400 000×. Scale bar is 20nm.
