# Supplementary material for: A prospective CSFV-PCV2 bivalent vaccine effectively protects against classical swine fever virus and porcine circovirus type 2 dual challenge and prevents horizontal transmission
Source: Vet Res. 2023 Jul 11;54:57. doi: 10.1186/s13567-023-01181-x (PMC10337183; doi:10.1186/s13567-023-01181-x)
Supplement: Supplementary file 2 — Additional file 2. Profile of PCV2 antigen and antibody levels as well as CSFV antigen and the dynamics of CSFV-specific antibodies in SPF pigs (Groups A, B, and C). The PCV2 and CSFV viral loads were detected by real-time PCR and expressed as copies/µL. The PCV2-specific antibody level was detected by an SLK105 kit (BioChek BV, Reeuwijk, The Netherlands), and the data were expressed as the S/P ratio. According to the kit’s protocol, serum samples with an S/P ratio greater than 0.50 were considered positive. In addition, the CSFV-specific antibody level was evaluated using the IDEXX CSFV Ab test kit (IDEXX Laboratories Inc., Liebefeld, Switzerland). The results were expressed as the blocking percentage, and according to the manufacturer, serum samples with a blocking percentage greater than 40% were considered positive. [file 13567_2023_1181_MOESM2_ESM.docx]

**Additional file 2 Profile of PCV2 antigen and antibody level as well as CSFV antigen and the dynamic of CSFV-specific antibody in SPF pigs (groups A, B, and C).** The PCV2 and CSFV viral load were detected by real-time PCR and expressed as copies/µL. The PCV2-specific antibody level was detected by An SLK105 kit (BioChek BV, Reeuwijk, The Netherlands) and the data was expressed as S/P ratio. According to the kit’s protocol, serum sample with S/P ratio greater than 0.50 are considered as positive. Besides, the CSFV-specific antibody level was detected using the IDEXX CSFV Ab test kit (IDEXX Laboratories Inc., Liebefeld, Switzerland). Results were expressed as the blocking percentage and according to the manufacturer, serum samples with a blocking percentage greater than 40% are positive. Data are present as mean ± standard error of the mean.

| **Items** | **Weeks of age** | | **Group A**  **(Bivalent vaccine)** | **Group B**  **(Placebo)** | **Group C**  **(Sentinel pigs)** |
| --- | --- | --- | --- | --- | --- |
| PCV2 viral load  (copies/µL) | | 6 | 1.32 ± 0.33 | 1.23 ± 0.27 | 0.36 ± 0.13 |
|  |  | 12 | 0.60 ± 0.50 | 0.84 ± 0.16 | 1.05 ± 0.82 |
| PCV2-specific antibody level  (S/P ratio) | | 6 | 2.10 ± 0.54 | 2.22 ± 0.33 | 2.04 ± 0.57 |
|  |  | 12 | 1.90 ± 0.60 | 0.73 ± 0.56 | 0.82 ± 0.35 |
| CSFV viral load  (copies/µL) | | 6 | 0 | 0 | 0 |
|  |  | 12 | 0 | 0 | 0 |
| CSFV-specific antibody level  (blocking percentage) | | 6 | 0 | 0 | 0 |
|  |  | 9 | 58.80 ± 5.84 | 0 | 0 |
|  |  | 12 | 73.36 ± 2.87 | 0 | 0 |
